# W-doped TiO_2_ nanoparticles with strong absorption in the NIR-II window for photoacoustic/CT dual-modal imaging and synergistic thermoradiotherapy of tumors

**DOI:** 10.7150/thno.33574

**Published:** 2019-07-09

**Authors:** Ke Gao, Wenzhi Tu, Xujiang Yu, Farooq Ahmad, Xiannan Zhang, Weijie Wu, Xiao An, Xiaoyuan Chen, Wanwan Li

**Affiliations:** 1 State Key Lab of Metal Matrix Composites, School of Materials Science and Engineering, Shanghai Jiao Tong University, 800 Dongchuan Road, Shanghai 200240, China; 2 The Comprehensive Cancer Center, Shanghai General Hospital, Shanghai Jiao Tong University School of Medicine, Shanghai 201620, China; 3 Laboratory of Molecular Imaging and Nanomedicine (LOMIN), National Institute of Biomedical Imaging and Bioengineering (NIBIB), National Institutes of Health (NIH), Bethesda, Maryland 20892, United States

**Keywords:** W-doped TiO_2_ nanoparticles, second near-infrared (NIR-II) window, multi-model imaging, thermoradiotherapy

## Abstract

Multifunctional nanomaterials that have integrated diagnostic and therapeutic functions and low toxicity, and can enhance treatment efficacy through combination therapy have drawn tremendous amounts of attention. Herein, a newly developed multifunctional theranostic agent is reported, which is PEGylated W-doped TiO_2_ (WTO) nanoparticles (NPs) synthesized via a facile organic route, and the results demonstrated strong absorbance of these WTO NPs in the second near-infrared (NIR-II) window due to successful doping with W. These PEGylated WTO NPs can absorb both NIR-II laser and ionizing radiation, rendering them well suited for dual-modal computed tomography/NIR-II photoacoustic imaging and synergistic NIR-II photothermal/radiotherapy of tumors. In addition, the long-term in vivo studies indicated that these PEGylated WTO NPs had no obvious toxicity on mice in vivo, and they can be cleared after a 30-day period. In summary, this multifunctional theranostic agent can absorb both NIR-II laser and ionizing radiation with negligible toxicity and rapid clearance, therefore it has great promise for applications in imaging and therapeutics in biomedicine.

## Introduction

Cancer is a leading threaten to human health. Currently available clinical treatments 1, 2, such as surgery, radiotherapy, and chemotherapy, typically induce significant side effects 3. Therefore, noninvasive tumor therapies with high treatment efficacy consistently attract a lot of attention 4, 5. Multifunctional nanomaterials have drawn more and more interest and have been rapidly developed and employed in cancer diagnostics and therapeutics recent years 6, 7. The ability of a single structure or particle to display multiple functions (nanotheranostics) 8 enables nanomaterials to be widely used in imaging 9, diagnosis 10, and treatment of cancers 11. Moreover, single systems that can also provide synergistic therapeutic approaches 7, 12 in addition to multi-modal imaging 13-15 have great potential for developing high efficiency cancer theranostics.

Radiotherapy (RT) that employs x- or γ-rays to destroy cancer cells with no depth restriction is widely used in the clinic 16-18. However, the side effects of irradiation on normal tissues 19 and low treatment efficacy against hypoxic cancer cells 20 limit the applications of RT. In recent years, nanomaterials with high-Z elements, such as gold 21, iodine 22, bismuth 23, tungsten 24 and rare earth elements 25, have been demonstrated as excellent radiosensitizers, which can concentrate radiation in tumors, and enhance the efficacy of RT while reduce possible associated side effects 26. Meanwhile, the combination of RT and photothermal therapy (PTT) can significantly increase the therapeutic efficiency of each alone due to the advantages of these methods and the compensation for the shortcomings of each other 27, 28. Tumor hyperthermia promotes blood flow and thus improves the oxygen status of cells 29. However, RT can kill tumor cells out of the range of PTT 16. Moreover, employment of a radiosensitizer is another way of reducing side effects as it allows the dose of radiation during RT treatment to be decreased 17, 30. Therefore, a combination of PTT absorption agent in the NIR-II and a radiosensitizer to decrease radiation doses is highly desired, especially for tumors in deep locations.

PTT utilizes the hyperthermia caused by NIR absorption to ablate tumors and is a highly localized and minimally invasive method 31. Importantly, employment of nanomaterials with high photothermal conversion efficiency in the first NIR window (NIR-I, 650-980 nm) could enhance therapeutic effects 7, 32-36. However, these effects are limited by the location depth of the tumors, since the laser intensity is inevitably attenuated as the tumor depth increases. Although lasers in the second NIR window (NIR-II, 1000-1700 nm) penetrate deeper and have higher maximum permissible exposures (MPEs) 37, there are fewer reports focusing on PTT in the NIR-II 38-40. Photoacoustic imaging (PAI) is a newly emergent diagnostic technique. By detecting the ultrasound caused by laser pulses, PAI combines the advantages of traditional ultrasound and optical imaging and has deeper tissue penetration and enhanced spatial resolution 41, 42. Due to its excellent imaging capabilities, PAI has been applied in molecular, cellular, vascular and tumor imaging 43-46. Moreover, the specificity and penetration depth of PAI could be further improved through exogenous contrast agents, including small molecule dyes 47-49, inorganic NPs 35, 50-52, and organic NPs 53-55. The PAI contrast is mainly determined by the optical absorption coefficient which depends on the wavelength of light; therefore, the PTT agents with strong NIR range absorption can be utilized as contrast agents for PAI. Recently, PAI in the NIR-II range has attracted increasing attention due to its stronger penetration ability and higher signal-to-background ratio compared with PAI in the NIR-I 38, 56-58. Therefore, development of contrast agents with intense absorption in NIR-II is essential for PAI, as well as PTT.

Recently, several types of nanomaterials including noble metal nanomaterials 59, 60, polymer nanocomposites 38, 58 and transition metal sulfide/oxide semiconductors 39, 51, have been reported to respond to NIR-II window as potential photothermal nanomaterials. Semiconductors show significantly different optical bandgaps and free carrier amounts via tuning doping or adjusting stoichiometric ratios, both of which dramatically contribute to semiconductor absorption in the NIR region. As a typical semiconductor, anatase TiO_2_ has a wide bandgap energy of 3.2 eV and strong ultraviolet (UV) absorption at around 380 nm. Boron 61, carbon 62, nitrogen 63-65, S 66, Co 67, W 68, 69, Fe 70 and rare earth elements 71, 72 have typically been used as dopants to attenuate the band-gap energy and expand the photoabsorption range of TiO_2_ in photocatalysis. Recently, Nb-doped TiO_2_ nanocrystals with strong absorption bands in the NIR range, resulted from efficient localized surface plasmon resonances, were obtained due to the considerable free electrons originated from the efficient incorporation of Nb^5+^ ions 73 and can act as excellent nanoagents for PTT in the NIR-II window. Meanwhile, because of the high atomic number of tungsten, tungsten-based nanomaterials have been widely explored as potential contrast agents for computed tomography (CT) 74-76 and radiosensitizers for RT 24. All these lead us to develop multifunctional W-doped TiO_2_ (WTO) nanoparticles (NPs) based on their hypothesized strong absorption band in the NIR range caused by the incorporation of W6+ ions and potential use as high efficiency PTT and PAI contrast agents. Moreover, the presence of tungsten inside NPs may allow utilization as CT imaging contrast and RT agents. So far there are no studies and reports on WTO NP photothermal and radiation properties, and potential applications in multimodel imaging, as well as other biomedical applications.

Herein, a novel WTO NP-based theranostic agent was successfully developed. This theranostic agent has strong absorption property in the NIR-II window, rendering it a potential agent for both dual-modal CT/NIR-II PAI and synergistic PTT/RT of cancer. First, a facile organic route of synthesizing WTO NPs was proposed. The PEGylated WTO NPs product exhibited a strong broad absorption ranging from ultraviolet to NIR wavelengths. A photothermal agent driven by 1064-nm laser with a high photothermal conversion efficiency of 44.8% and excellent photostability was found to be a successful approach. The WTO NPs also showed application potential as in vivo contrast agents for CT and NIR-II PAI based on their great X-ray attenuation coefficient of W and strong absorption in the NIR-II range. PEGylated WTO NPs were also combined with RT and PTT for tumor treatment in tumor-bearing mice and showed dramatic synergistic effects. Furthermore, PEGylated WTO NPs displayed both low toxicity and excellent biocompatibilities* in vivo*. Our results highlight the potential future applications of WTO NPs as well as other multifunctional nanomaterials in cancer diagnostics and therapy based on the doped semiconductor NPs for cancer theranostics.

## Results and Discussion

### Synthesis and Characterization of Nanoparticles

**Figure 1:**
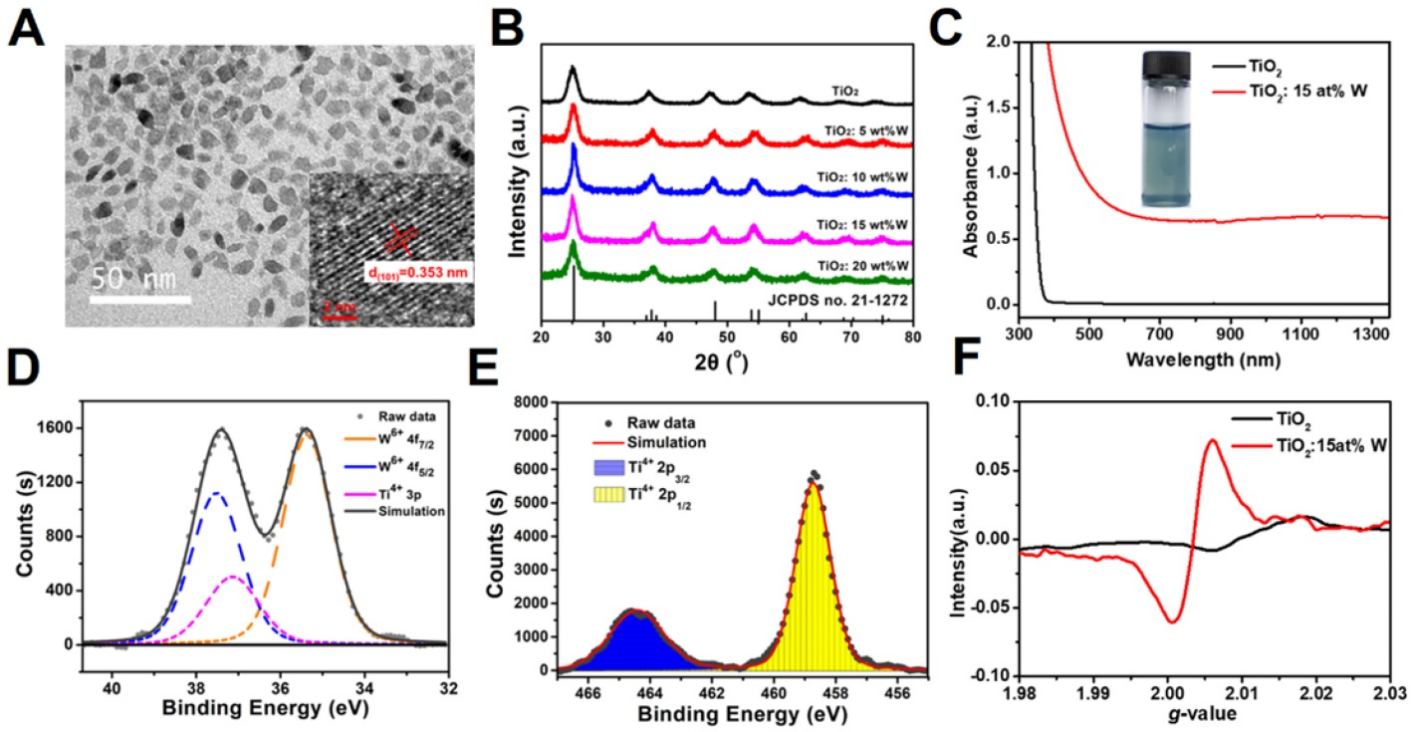
(A) TEM image of TiO_2_: 15 at% W. Inset shows the HRTEM image. (B) XRD patterns of TiO_2_ and W-doped TiO_2_. (C) UV-vis-NIR spectra of TiO_2_ and TiO_2_: 15 at% W dispersed in chloroform (Ti concentration = 600 μg mL^-1^). Inset shows the color. (D, E) XPS fitting spectra of (D) W4f and (E) Ti2p. (F) EPR spectra of TiO_2_ and TiO_2_:15 at% W.

WTO NPs were successfully synthesized through a facile modified organic route 73. Briefly, tetraethyl titanate [Ti(OC_2_H_5_)_4_] and tungsten (VI) chloride (WCl_6_) were heated to 290°C for 1 hour in an anhydrous and oxygen-free system consisting of 1-octadecanol, oleic acid, and 1-octadecene. Under these conditions, several WTO samples were doped with different amounts of W (TiO_2_: 5 at% W, TiO_2_: 10 at% W, TiO_2_: 15 at% W, and TiO_2_: 20 at% W) by changing the molar ratio of WCl_6_/Ti(OC_2_H_5_)_4_ in the precursor solution. Undoped TiO_2_ NPs were also prepared in the absence of WCl_6_ precursor for comparison. The morphologies of these samples were assessed by transmission electron microscopy (TEM). As shown in **Figure 1a** and **Figure S1**, the as-prepared TiO_2_ NPs (15 at% W) were not uniform in shape, which included primarily “rice grains” with lengths of 9.1 ± 2.2 nm and diameters of 5.7 ± 1.4 nm. In our work, we tried to dope WTO nanoparticles using W precursor concentration as high as possible to ensure enough W inside the WTO nanoparticles for effective CT imaging and RT. As shown in **Figure S1**, with the increase of doping concentration of W, the morphology of the nanoparticles changes gradually. When the W doping concentration is 15 at%, the uniformity of resultant WTO NPs can be maintained. However, the uniformity of WTO NPs becomes poor when the W doping concentration is higher than 15 at%. Therefore, WTO NPs with W doping concentration of 15 at% were used for further investigation. High-resolution TEM (HRTEM) images of TiO_2_: 15 at% W (inset of **Figure 1a**) reveal lattice fringes with an interplane spacing of 0.358 nm corresponding to the (002) plane of anatase TiO_2._ All the samples had similar X-ray powder diffraction (XRD) patterns and yielded six peaks at 25.3°, 37.8°, 48.0°, 53.9°, 55.1°, and 62.7° corresponding to the (101), (004), (200), (105), (211), and (204) planes of anatase TiO_2_ (JCPDS no. 21-1272), respectively (**Figure 1b**, a). These results indicate W-doping does not change the crystal phase of anatase TiO_2_ 77, which may be attributed to the similar ionic radii of W^6+^ (0.60 Å ) and Ti^4+^ (0.605 Å)^78^. Moreover, as shown in **Figure S2**, the distributions of Ti, W, and O in the TiO_2_: 15 at% W NPs were homogeneous, indicating successful doping with W. The chemical states of W and Ti in the TiO_2_: 15 at% W NPs were assessed by XPS, which confirmed the valence of W was +6 **(Figure 1d)** and Ti was +4 **(Figure 1e)**.

UV-vis-NIR spectra were applied to characterize the optical properties of these samples. As shown in **Figure 1c**, the intrinsic TiO_2_ NPs dissolved in chloroform only absorbed ultraviolet light, while the deep blue WTO NP solution exhibited a strong and continuous absorption from visible light to NIR (>1350 nm) without yielding any sharp peaks outside of the intense UV absorption. The expansion of photoabsorption could be attributed to the increase in free carrier concentration as revealed by the electron paramagnetic resonance (EPR) spectra **(Figure 1f)**. Specifically, the peak around 2.01 eV was related to the WTO NP electrons. The doped W^6+^ replaced Ti^4+^, releasing electrons to form free carriers and additional electronic states between the conduction and valence bands of the TiO_2_ and thus expanding the absorption spectrum from the ultraviolet to NIR range. Functional groups on the surface of the NPs are important for biomedical applications. Coating of WTO NPs with a hydrophilic layer was performed at room temperature via sonication with a DSPE-PEG_5000_ solution. The surface modification was investigated by FTIR and the intensities of the C-O-C stretch band (1101 cm^-1^) displayed a distinct increase after coupling based on the DSPE-PEG_5000_ peak **(Figure S3)**. The morphology of the PEGylated WTO NPs was confirmed by TEM **(Figure S1b)**, where little change was observed compared to those without PEG coating. Moreover, the PEGylated WTO NPs were also proved to have excellent stability and dispersibility in different kinds of physiological media **(Figure S4)** with hydraulic radii of ~47 nm **(Figure S5)**, suggesting that NPs have been successfully modified for potential further use both *in vitro* and *in vivo*. In addition, the aqueous PEGylated WTO solution maintained the optical absorption properties and the absorption intensity increased as the NP concentration increased **(Figure S6)**.

### *In vitro* Photothermal Performance

**Figure 2:**
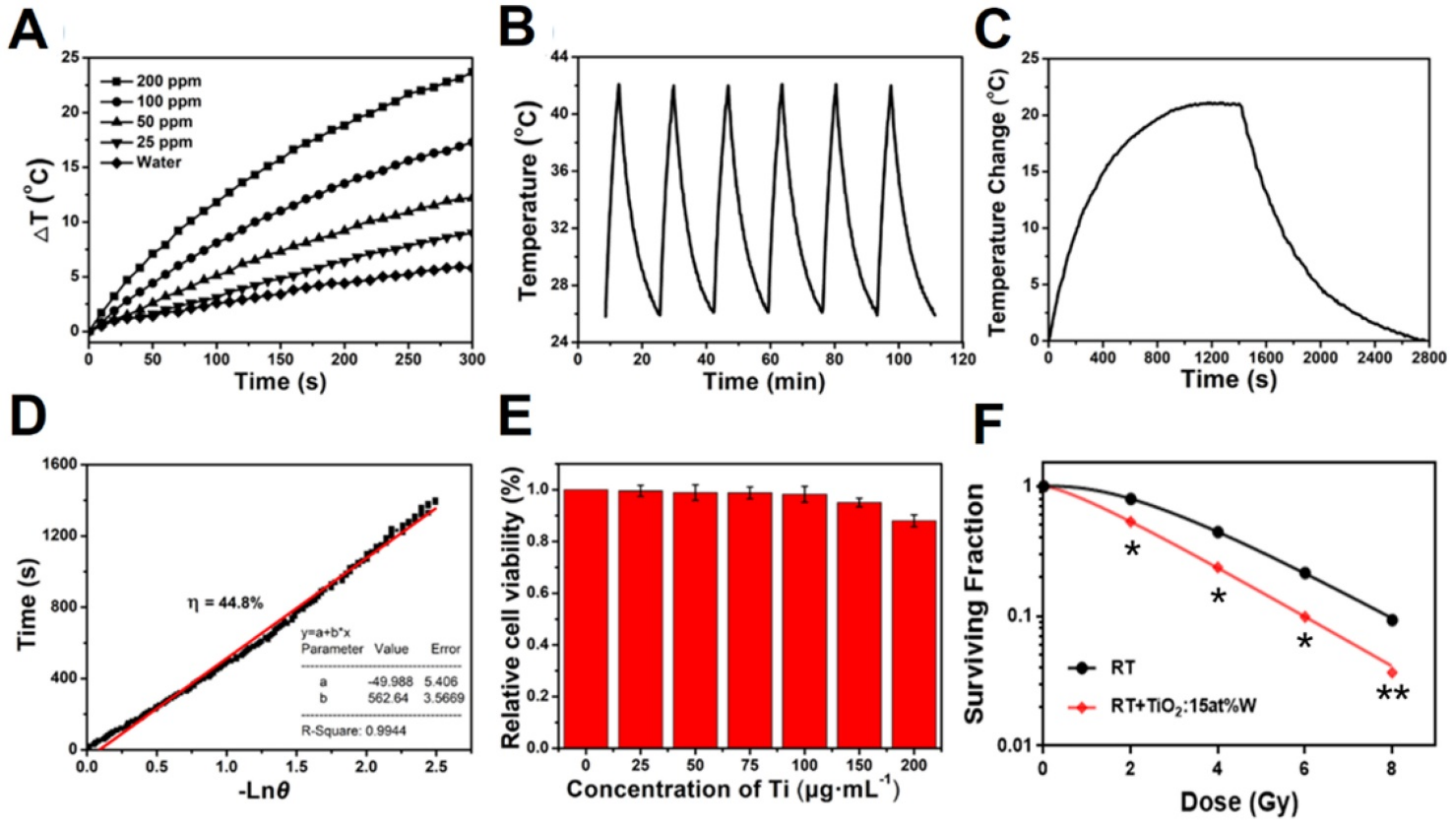
(A) Temperature changes of WTO NP solution with different concentrations of Ti under 1064-nm laser irradiation at 1 W cm^-2^ for 5 minutes. (B) Temperature changes of a WTO NP solution with a Ti concentration of 200 ppm over six cycles under 1064-nm laser irradiation at 1 W cm^-2^. (C) Temperature profile of a WTO NP solution with a Ti concentration of 100 ppm over 2800 s under 1064-nm laser irradiation at 1 W cm^-2^ for about 1400 s. (D) Calculation of time constant for heat transfer from the system using linear regression of the cooling profile. (E) Relative viabilities of 4T1 cells treated with WTO NPs with different concentrations of Ti for 24 h were evaluated using CCK-8 assay. (F) Inhibitory effect of WTO NPs (0 or 100 ppm of Ti) on 4T1 cells under different irradiation dosages. (*p<0.05,**p<0.01)

The PEGylated WTO NPs showed a strong and continuous absorption from visible light to NIR (>1350 nm), exhibiting no sharp peaks, which led us to apply a common laser with a 1064-nm wavelength to trigger the NIR-II PTT. The photothermal properties of the NPs were first assessed via examination of the temperature variation in NP solution containing different concentrations of Ti (0, 25, 50, 100, and 200 ppm) irradiated using a 1064-nm laser. The temperature variation for 1 mL NP solution in plastic tubes was recorded during the irradiation (300 seconds, 1 W cm^-1^). **Figure 2a** shows that the water temperature increased by only 5°C and the PEGylated NP solution exhibited a concentration-dependent temperature enhancement. As the concentration of Ti increased from 25 to 200 ppm, the final temperature change (ΔT) increased from ~9 to ~24 °C. The stability of the photothermal conversion of the WTO NPs was evaluated next using a cycle experiment. WTO NP solution (1 mL, 100 ppm) were irradiated with a 1064-nm laser at 1 W cm^-1^ for 5 minutes and then the laser was turned off to let the solution cool to 26°C. As shown in **Figure 2b**, the change in temperature was almost the same during each laser on-off cycle, indicating the WTO NPs had excellent photothermal stability. Photothermal conversion efficiency (*η*) is an important parameter for evaluating photothermal agents and can be calculated as described previously 79. The *η* value of the PEGylated WTO NPs was 44.8% at 1064 nm **(Figure 2c and 2d)**, which is higher than the reported *η* values of other PTT agents in the NIR-II window 39, 59, 80, 81.

### *In vitro* Cytotoxicity Assay and Cellular Uptake

TiO_2_ is an environmentally friendly material and white TiO_2_ and black H-TiO_2_ NPs are considered to also have low toxicity and good biocompatibility 82. Here, we investigated the cytotoxicity of PEGylated WTO NPs with a CCK-8 assay. As can be seen in **Figure 2e**, PEGylated WTO NPs caused no significant 4T1 cell cytotoxicity as the relative viability was higher than 85% even with treatment of 200 μg mL^-1.^Ti. Moreover, **Figure S7** shows the intracellular uptake of PEGylated WTO NPs, the cellular internalization was enhanced with the increasing incubated time, and the concentration of Ti in cells reached a relatively saturated level after 8 hours of incubation. The characteristics of PEGylated WTO NPs including high* η* value, low cytotoxicity, great photothermal stability, and strong penetration capability in tissue make them promising photothermal agents for application in NIR-II window.

### Clonogenic Assays

After demonstrating the ability of PEGylated WTO NPs to trigger PTT, a clonogenic survival assay was carried out to determine whether PEGylated WTO NPs can enhance RT efficacy. As shown in **Figure 2f**, WTO NPs enhanced radiotherapy when the concentration of W was 100 ppm. The SF_2_ value, which indicates the cell survival fraction when the radiation dose is 2 Gy, was 0.553 for the combination treatment group and 0.799 for the group lacking WTO NPs **(Table 1)**. The SER10 was 1.323 when the SF value was 0.1, which is higher than that of AuNPs (1.19) and similar to that of paclitaxel (1.32) 83, demonstrating significant radiosensitization by PEGylated WTO NPs.

### *In vitro* Photothermal Therapy and Radiation Therapy

To test the therapeutic efficiency of PEGylated WTO NPs *in vitro,* different therapeutic experiments in cellular levels were performed. As shown in** Figure S8**, NIR alone and X-ray alone treatment decreased 4T1 cell viability to 93 and 85%, respectively, while the PTT effect and the RT effect of WTO nanoparticles can result in the obvious decrease of the cell viability to 62 and 51%. When the PTT effect combined with RT effect, it was found that cell viability substantially decreased to 21%, confirming the considerable WTO nanoparticles enhanced RT/PTT synergistic effect *in vitro*, which encouraged us to further study the synergistic therapeutic effects of photothermal and radiotherapy through animal models.

### *In vivo* Blood Circulation and Tumor Accumulation

Motivated by the experimental results *in vitro*, we next examined the pharmacokinetic behavior and tumor accumulation pattern of PEGylated WTO NPs (3 mg/mL, 200 µL) in tumor-bearing mice using intravenous (i.v) administration. For the pharmacokinetic study, we took blood samples at different time points, followed by inductively coupled mass spectrometer (ICP-MS) analysis to measure the amount of Ti. As shown in **Figure S9**, the content of PEGylated WTO NPs in the blood of mice gradually decreased with time. And the half-decay time of PEGylated WTO NPs circulating in the blood was approximately 1.28 hours, indicating that the WTO NPs modified by mPEG-DSPE possess well biocompatibility and could cycle in the blood for a period of time, which is conducive to the enrichment of nanomaterials at tumor site. Then we further studied the biodistribution of PEGylated WTO NPs on 4T1 tumor bearing mice. As shown in **Figure S10**, 24h after tail-vein injection, NPs mainly concentrated in spleen, liver and kidney, the enrichment percentage of PEGylated WTO NPs at the tumor site is about 5% injected dose per gram (ID g^-1^) due to the enhanced permeability and retention (EPR) effect.

### *In vitro* and *In vivo* X-ray Computed Tomography

**Figure 3:**
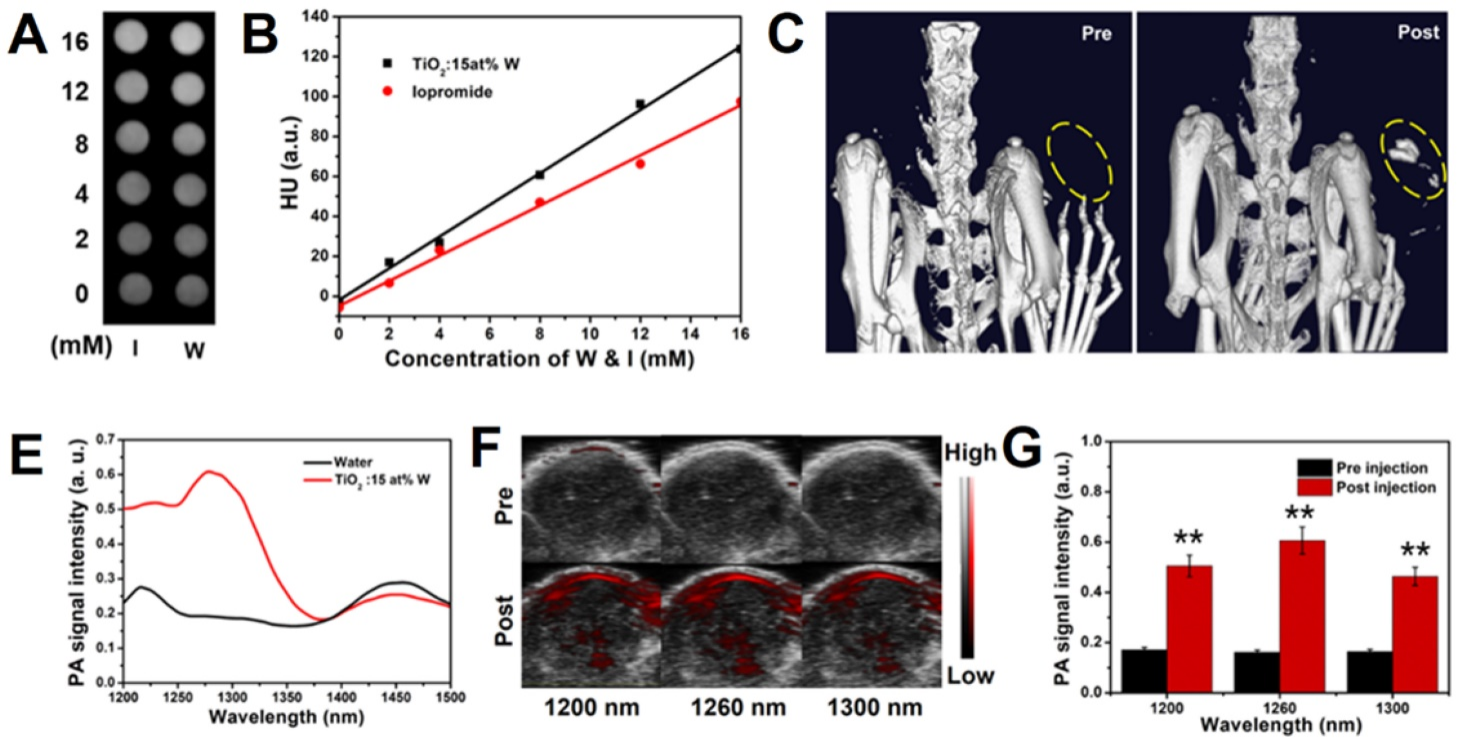
(A) *In vitro* CT images of iopromide (upper row) and WTO NP solution (lower row) of different concentrations. (B) HU values of various concentrations of WTO NP and iopromide solution. (C) *In vivo* CT images of mouse pre- (left) and post-intratumoral (i.t.) injection (right) with WTO NP solution. (D) *In vitro* PA signal intensity of WTO NP solution (0.5 mg mL^-1^ of Ti) and water from 1200 to 1500 nm. (E) *In vivo* ultrasonic PA composite images pre- (upper row) and post- (lower row) i.t. injection of WTO NP solution at 1200, 1260, and 1300 nm. (F) *In vivo* PA signal intensity of tumors pre- (black) and post-i.t. injection (red) of WTO NP solution( **p<0.01).

The dramatic photoelectric and NIR absorbance of the W atoms in the PEGylated WTO NPs under the radiation of X-ray them to be potentially applied in tumor PTT, PA/CT dual-modal imaging, and sensitizing RT. To evaluate the performance of PEGylated WTO NPs in CT imaging as a contrast agent *in vitro*, a commonly used CT contrast agent of iopromide in clinic was employed for comparison. As shown in **Figure 3a**, both the WTO and iopromide solution exhibited concentration-dependent signal enhancement, but the PEGylated WTO group showed a higher signal intensity than the corresponding iopromide group when the W and I concentrations were equal. Quantification of signal intensity revealed the slopes of the CT values of the PEGylated WTO NPs and iopromide were ~7.9 and ~6.3, respectively **(Figure 3b)**. The beneficial effect of using PEGylated WTO NPs in CT imaging *in vitro* encouraged us to assess their use with CT imaging in 4T1 tumor-bearing mice. As illustrated in **Figure 3c**, the CT value of the tumor region (yellow dashed circle) for the tumor to be visualized before i.t. injection. Upon injection of 30 μL of PEGylated WTO NPs with 6 mg mL^-1^ of W, tumor displayed clear contrast with a white area. This observed increase suggests PEGylated WTO NPs could be used as an effective CT contrast agent.

### *In vitro* and *In vivo* Photoacoustic Imaging

PAI employs laser to acoustically visualize biological tissues, therefore it possesses unique spatial resolution and high imaging depth, especially in the NIR-II window. For NIR-II PAI *in vitro*, we collected photoacoustic images of PEGylated WTO solution (0.5 mg mL^-1^) and deionized water in micro polyurethane tubing from 1200 to 1500 nm. We quantified the signal intensity of the photoacoustic images from 1200 to 1500 nm **(Figure 3d)** and found the PA signal of the PEGylated WTO NPs solution was 2-3 times stronger than that of water with a wide wavelength range between 1200 and ~1325 nm. As shown in **Figure S11**, the PEGylated WTO NPs displayed strong PA signals at 1200, 1260, and 1300 nm, while the water group yielded weak signals that were almost not visible in images taken at the same wavelength. The *in vivo* tumor PAI efficiency was assessed by calculating the PA values of the mice bearing 4T1 tumors after i.t. administration of PEGylated WTO NPs **(Figure 3e)**. The mice bearing 4T1 tumors were PA imaged prior to i.t. injection of PEGylated WTO NPs as a control and there was no obvious photoacoustic signal present except for a low signal from the skin. By contrast, intense photoacoustic signals were detected from the tumor at 1200, 1260, and 1300 nm after injection. The signal quantization results shown in **Figure 3f** demonstrate the PA signals of the post-injection group were approximately 2.95- (1200 nm), 3.75- (1260 nm), and 2.82-time (1300 nm) that of the pre-injection group, indicating PEGylated WTO NPs are also suitable for PAI as a contrast agents in a wide range of NIR-II wavelengths.

### NIR-II Photothermal and Radiotherapy *In Vivo*

**Figure 4:**
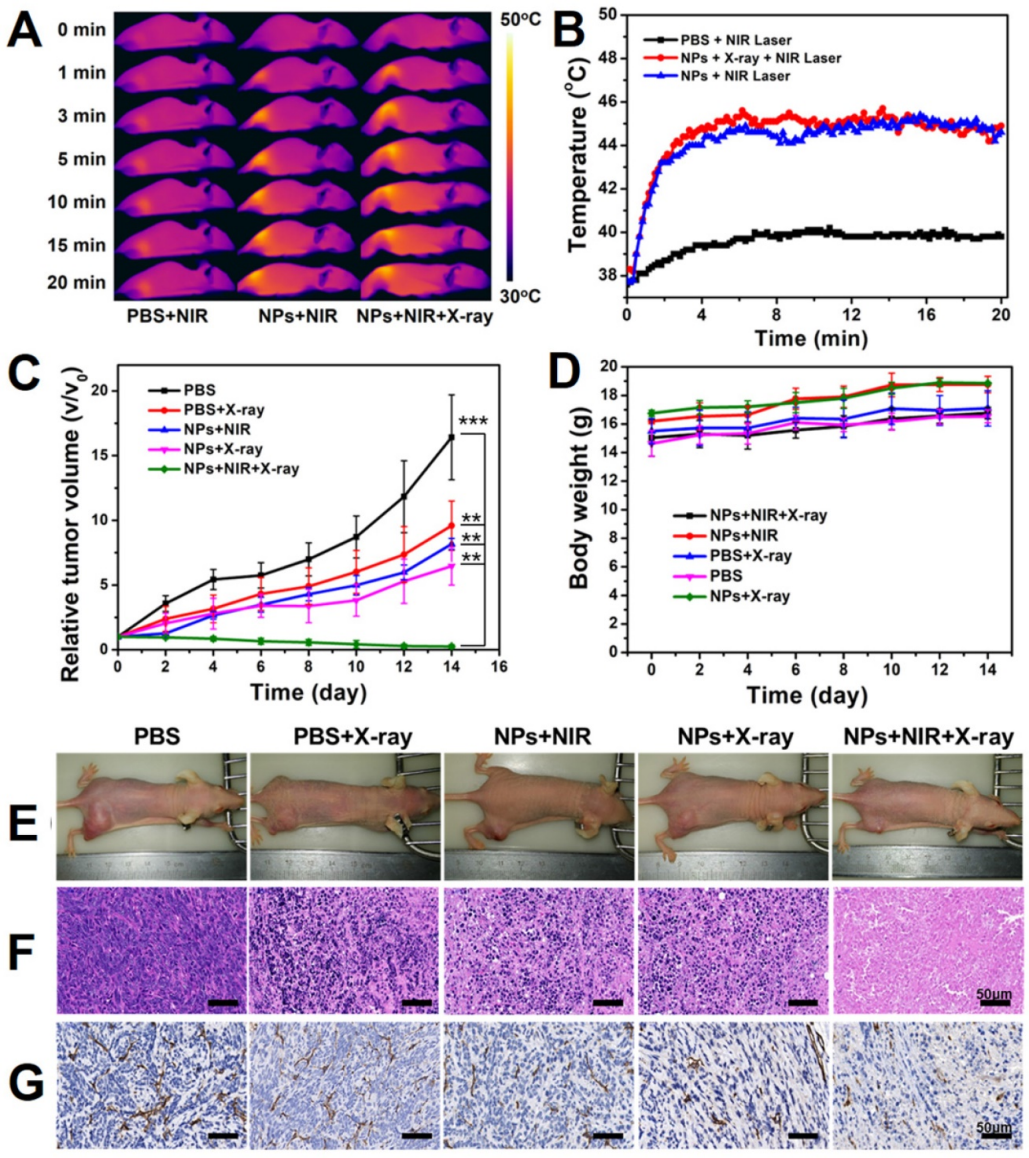
(A) Temperature variation on the surface of tumors in mice under laser irradiation of 1064 nm after i.v. injection with PBS or WTO NP solution. (B) Infrared images of mice under 1064 nm laser irradiation after i.v. injection with PBS or WTO NP solution. (C) Tumor volume and (D) body weight variation curves of mice in each group. (E) Photographs of mice 14 days after exposure to different treatments. (F) H&E and (g) CD31 staining of tumor sections 24 h after exposure to different treatments (**p<0.01, ***p<0.005).

We next evaluated the *in vivo* anti-cancer efficacy of the PEGylated WTO NPs in a 4T1 tumor-bearing mouse model. Mild tumor hyperthermia was induced by administering a low dose sample and then combination treatment of PTT/RT was applied to evaluate their effect. 4T1 tumor-bearing BALB/c nude mice were randomly divided into five groups (n=5) to study different treatments. For the PTT, we recorded infrared videos of mice during NIR-II laser irradiation **(Figure 4a)**. After quantification of the infrared images, we illustrated temperature variation on the tumor surfaces **(Figure 4b)**. Under a 1064-nm laser irradiation at 0.5 W cm^-2^, the temperature of the NP-injected groups increased to ~45°C in 5 min and was maintained for 15 min, which guaranteed mild tumor hyperthermia, while the PBS-injected group exhibited only a 40°C maximum temperature **(Figure 4a and b)**. A similar phenomenon was observed for the groups treated with radiotherapy, but not NIR laser irradiation, and tumor growth was only slightly inhibited. Notably, the growth rate of the group treated with NP injection and X-ray irradiation was lower than that of the group receiving PBS injection and X-ray irradiation, confirming enhancement of RT by the PEGylated WTO NPs. Compared with the groups only treated with PTT or RT, the group receiving the combination therapy of NP injection and NIR-II laser and X-ray irradiation exhibited significant inhibition of tumor growth. By contrast, the control tumors displayed uninhibited growth in the PBS group **(Figure 4c and e)**.

To identify the changes inside the tumors, we stained tumor slices using hematoxylin and eosin (H&E, see** Figure 4f**) and also incubated with anti-CD31 antibody **(Figure 4g)**. We observed remarkable damage of tussues and significant tumor angiogenesis suppression in PTT/RT combination treatment group only, consistent with the anti-cancer effect of the PTT/RT combination treatment mentioned above. Moreover, no significant loss of body weight and death of mice were observed, and all of the mice behaved normally **(Figure 4d)**. Our results demonstrate that PEGylated WTO NPs can be used as excellent agents for NIR-II PTT and RT, furthermore, when these therapies were used together, there are synergistic effects against tumors.

### Blood Panels and Histological Examinations *In vivo*

**Figure 5:**
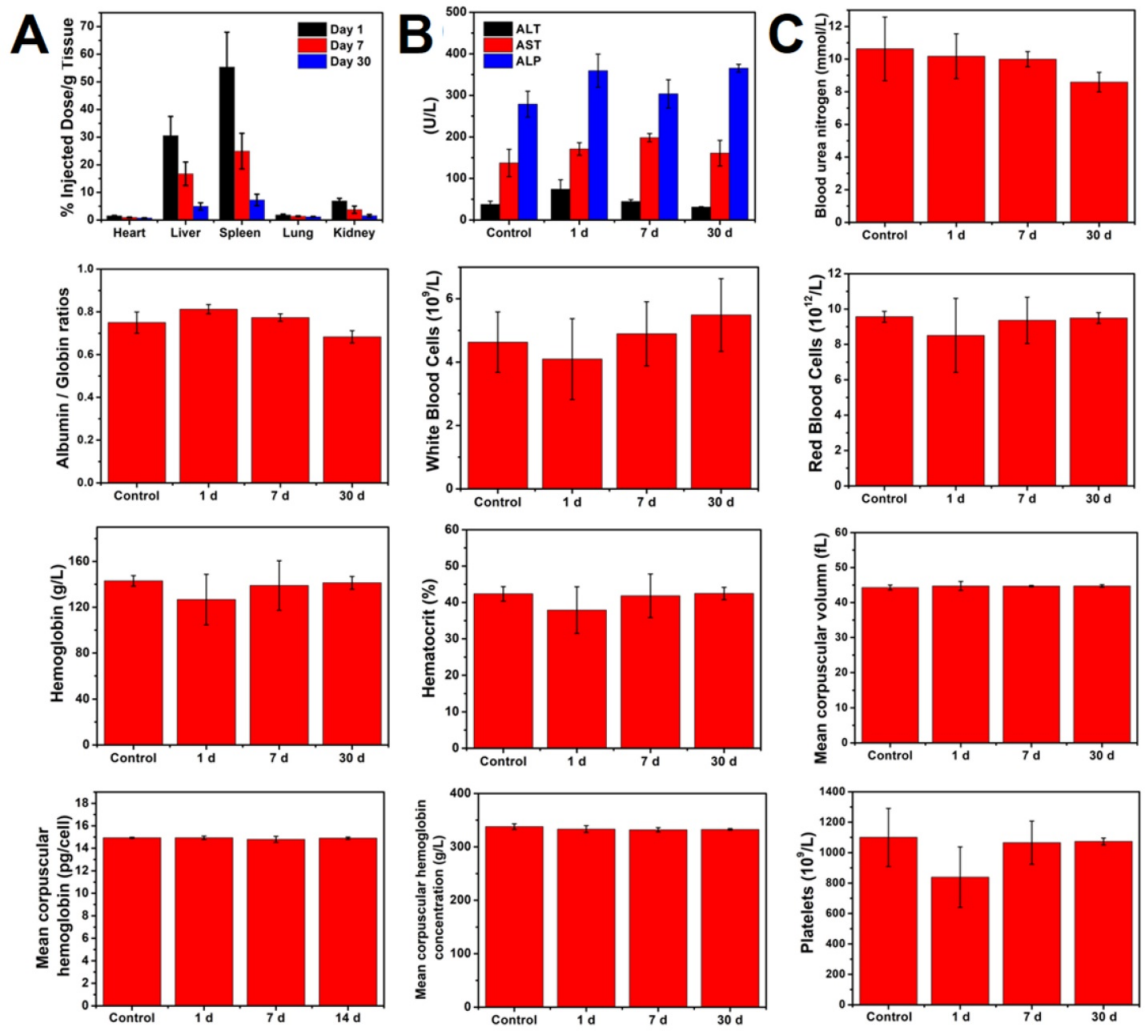
(A) Biodistribution of WTO NPs in organs of mice sacrificed at different time points. (B) Blood biochemistry ALT, AST, and ALP analysis of mice sacrificed at different time points. (C) Hematology analysis of eight blood panel parameters of mice sacrificed at different time points.

Considering that accumulation of NPs in vivo might induce unwanted side effects or toxicity after a long-term treatment, we next evaluated the potential long-term toxicity of PEGylated WTO NPs *in vivo*. After intravenous injection of PEGylated WTO NPs for 1, 7, and 30 days, mice were sacrificed, complete blood panel and blood biochemistry analyses were performed and major organs (liver, heart, lungs, spleens and kidneys) were harvested for ICP-AES and H&E staining. The complete blood panel measured the albumin/globin ratio (A/G), blood urea nitrogen (BUN), red blood cell (RBC), white blood cell (WBC), hematocrit (HCT), hemoglobin (HGB), mean corpuscular hemoglobin concentration (MCHC), mean corpuscular volume (MCV), mean corpuscular hemoglobin (MCH), and platelet count (PLT). (**Figure 5a**). Although the BUN, WBC, RBC, HGB, HCT, and PLT levels decreased slightly 1 day after administration of PEGylated WTO NPs, the values returned to normal levels by days 7 and 30. All other parameters were normal. The blood chemistry analysis including aspartate aminotransferase (AST), alkaline phosphatase (ALP), and alanine aminotransferase (ALT) was carried out and no abnormalities were detected compared with the control group, suggesting that the PEGylated WTO NPs caused no obvious liver toxicity (**Figure 5b**). In addition, neither visible inflammation nor organ damage was observed from the H&E staining images of tumors **(Figure S12)** from either the treatment and control groups. By measuring the Ti content in major organs by ICP-AES **(Figure 5c)**, we found the injected NPs mainly accumulated in the spleen and liver, while the heart, lungs, and kidneys displayed relatively low Ti accumulation levels. The Ti content decreased in all organs over time and became relatively low at 30 d post-injection. These results confirmed the low long-term toxicity and excellent biocompatibility of the PEGylated WTO NPs *in vivo*.

## Conclusions

In summary, we showed that PEGylated WTO NPs with diameters averaging ~10 nm could be facilely synthesized via an organic route. PEGylated WTO NPs had strong absorbance in the NIR-II window due to successful doping with W, therefore exhibiting excellent NIR-II photothermal properties and photostability. Following modification with DSPE-PEG5000, the resulting PEGylated WTO NPs became an excellent candidate agent for dual-modal CT/NIR-II PAI. In addition, PEGylated WTO NPs had the beneficial properties of absorbing both NIR-II laser radiation and ionizing radiation, as well as enhancing RT efficacy. We showed that these NPs can result in a dramatic synergistic effect on RT and PTT for tumor treatment *in vivo*. In contrast, treatment with RT or PTT alone can partially suppress tumor growth. The long-term clearance and toxicity results showed that PEGylated WTO NPs can be completely cleared within 30 days in mice without obvious toxicity. In summary, this promising theranostic agent with its excellent imaging and therapeutic properties coupled with its low toxicity and reasonable clearance has great promise for use in the field of biomedical science.

## Materials and Methods

### Materials

Tungsten (VI) chloride and 1-octadecanol were purchased from Macklin (Shanghai, China). Oleic acid and 1-octadecene were obtained from Sigma Aldrich. Titanium (IV) ethoxide was purchased from Alfa Aesar (UK). DSPE-PEG_5000_ was purchased from Shanghai Yudu Biotechnology (China). Acetone, hexane, and chloroform were purchased from Yonghua Chemical Technology (Jiangsu, China). Iopromide injection was purchased from Bayer Pharma AG (Germany). The 4T1 cells were provided by the Shanghai Center for Systems Biomedicine (Shanghai, China). A cell counting kit-8 (CCK-8) kit was obtained from BBI Solution (UK). Female BALB/c nude mice of 5~6 weeks old were obtained from Slac Laboratory Animal Center (Shanghai, China).

### Synthesis of WTO NPs

Under a vacuum at 110°C for 1 h, 20 mmol of 1-octadecanol was dissolved in a mixture of 1-octadecene (7 mL) and oleic acid (3 mL) in a 25 mL three-neck flask connected to a Schlenk line. Then, the solution was heated to 130°C under a nitrogen atmosphere and maintained for 30 min to reach an anhydrous, oxygen-free state. Next, 2 mmol of tetraethyl titanate and 0.3 mmol of tungsten (VI) chloride dissolved in 10 mL methanol was injected into the three-neck flask once the solution had cooled to 80°C. When the methanol had completely evaporated, the temperature of the system was raised to 290°C and maintained for 1 h. Acetone and n-hexane were then added to wash the precipitate and then the solution was centrifuged to obtain NPs, followed by redispersion in chloroform.

### PEGylation of Nanoparticles

Chloroform solution (2 mL) containing 10 mg of NPs was mixed with 5 mL chloroform solution containing 20 mg DSPE-PEG_5000_ in a 25-mL three-neck flask. After stirring in the presence of continuous nitrogen gas at room temperature overnight, the chloroform was evaporated. Deionized water (2 mL) was added to the flask to redisperse the NPs. After sonicating for 5 minutes, PEGylated NPs were completely dissolved in water and became hydrophilic. To remove large aggregates that possibly formed during surface modification, the aqueous solution was centrifuged at 3000 rpm for 10 min. The final product was stored at 4°C until use.

### *In vitro* Photothermal Property

To evaluate the photothermal performance of the NPs, 1 mL aqueous solution containing various concentrations of NPs was added to plastic tubes and then irradiated with a 1064-nm laser. Temperature variation and thermal photos were recorded with an FLIR E50 thermal camera.

### *In vitro* Cytotoxicity Assay and Cellular Uptake

The cytotoxicity of PEGylated NPs was evaluated using CCK-8 assays. The 4T1 cells were plated in 96-well plates at 7 × 10^3^ cells per well and incubated for 24 h. The culture media were then replaced with fresh culture media containing NPs and different concentrations of Ti (0, 25, 50, 75, 100, 150, and 200 μg mL^-1^) and incubated for another 24 h. Finally, cells were washed with fresh media to remove the NPs and cell viability was examined using a CCK-8 assay.

In order to study the ability of 4T1 cells to internalize WTO NPs, 4T1 cells were grown in six well plates (1 × 10^5^ cells/well) for 24 h. The culture media were then replaced with culture media containing WTO NPs with a Ti concentration of 100 ppm and the plates were then incubated at 37 °C for a series of time (0.5,1, 2, 4, 8, and 12 h). Following this incubation, cells were washed with PBS three times and released with cell dissociation buffer. Finally, cells were centrifuged and then digested by concentrated aqueous HNO_3_. The amount of Ti was then analyzed using ICP-MS.

### Clonogenic Assays

To measure the radiosensitization by NPs, two groups of 4T1 cells were plated in six-well plates at different densities (100, 200, 500, 1000, and 2000 cells/well) and incubated for 24 h. The culture media were then replaced with fresh media containing NPs and different concentrations of W (0 or 100 μg mL^-1^). After incubating for another 24 h, the plates were irradiated with different doses of X-rays (0, 2, 4, 6, and 8 Gy). Thereafter, the cells were incubated with fresh media for another 10 days under standard conditions. Finally, the surviving colonies were enumerated and used plating efficiency (PE), surviving fraction (SF), and sensitizer enhancement ratio (SER) were calculated.^84^ SER10 refers to the sensitizer enhancement ratio at 10% cell survival and is critical for evaluating the radiosensitization effect of a radiosensitizer.^83^, ^85^ The D_0_ and N values were calculated from the fitting results of a multitarget single-hit model, SF = 1-(1-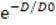)^N^.

PE =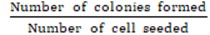 × 100%

SF = 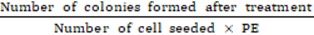 × 100%

SER = 
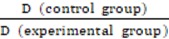


### *In vitro* Photothermal Therapy and Radiation Therapy

To test the therapeutic efficiency *in vitro*, 4T1 cells wre cultured in 96-well plates at a density of 7 × 10^3^ cells per well for 24 h. After the cells had grown to the full bottom of the plates, the cells were set to eight groups (control, X-ray, NIR-II, NPs, NPs+NIR, NPs+X-ray, NIR+X-ray and NPs+NIR+X-ray ). The meda were replaced by fresh meda or media containing WTO NPs with a Ti concetration of 100 ppm. Followed incubation for 8h, 4T1 cells were treated with 1064 nm laser (1 W cm^-2^, 10 minutes ) or X-ray (4Gy) respectively and then incubated for 24h. Finally, cells were washed with fresh media to remove the NPs and cell viability was examined using a CCK-8 assay.

### Animal Experiments

All animal operations were in accordance with Shanghai Jiao Tong University institutional regulations regarding animal use and care. Tumors were established by subcutaneously injecting of 2 × 10^6^ 4T1 cells (in 60 μL PBS) into their right legs of nude mice. The treatments were started when the tumor volume (volume = length × width^2^/2) reached approximately 60 mm^3^.

### *In vivo* Blood Circulation and Tumor Accumulation

A PBS solution of WTO NPs (200 μL, 3 mg mL^-1^ of Ti) was injected intravenously into healthy mice (n=5). Blood was then collected from the eyelids of mice at different time points (5 min ,0.25, 0.5, 1, 2, 4, 8, 12 and 24 h) after injection. Finally, samples were digested by aquous HNO_3_ before the amount of Ti was measured by ICP-MS.

4T1 tumor-bearing BALB/c mice were intravenously injected with 200 μL of WTO NP solution (3 mg mL^-1^ of Ti) and euthanized after 24 h, respectively (n = 5). Tumors and main organs were collected and then digested by concentrated aquous HNO_3_ overnight before the amount of Ti was measured by ICP-MS.

### *In vitro* and* in vivo* X-ray Computed Tomography

To evaluate the CT imaging performance of NPs* in vitro*, iopromide was used for comparison. NP solution (200 μL) containing different concentrations of tungsten or iodine (0, 2, 4, 8, 12, or 16 mM) were CT imaged with a GE light-speed VCT at 80 kV and 500 mA with a 0.625 mm slice. Iopromide solution containing the corresponding concentrations of I were set as the control.

For* in vivo* CT imaging, nude mice were anesthetized by intraperitoneal injection of pentobarbital sodium solution. Then CT images of the mice before and after intratumoral (i.t.) injection of WTO solution were acquired using the Quantum GX.

### *In vitro* and *in vivo* Photoacoustic Imaging

To evaluate the *in vitro* photoacoustic imaging performance of the NPs, we used micro polyurethane tubing to hold a NP solution with 0.5 mg mL^-1^ Ti and deionized water as a control. Then a multimode small animal ultrasound/photoacoustic imaging system was employed to collect both photoacoustic and ultrasonic images and then the photoacoustic signals were quantified by VevoLAB software at an excitation of 1200 to 1500 nm.

For* in vivo* photoacoustic imaging, nude mice were anesthetized and photoacoustic and ultrasonic images of tumors both before and after i.t. injection of 30 μL of PEGylated NPs with 1 mg mL^-1^ Ti were acquired using the multimode small animal ultrasound/photoacoustic imaging system.

### NIR-II Photothermal and Radiotherapy *in vivo*

For *in vivo* therapy assessment of NPs, 4T1 tumor-bearing BALB/c nude mice were randomly divided into 5 groups (n=5): (I) PBS injection, (II) PBS injection + X-ray, (III) NP injection + NIR laser, (IV) NP injection + X-ray, and (V) NP injection + NIR laser + X-ray. First, the mice in each group were intravenously injected with 200 μL of PBS or NP solution with a Ti concentration of 3 mg mL^-1^. After 24 h, groups III and V were treated with 1064-nm laser irradation (0.5 W cm^-2^, 20 minutes), while groups II, IV, and V were treated with X-rays (4 Gy). During the laser irradiation, an infrared thermal video of each mouse was recorded and the temperature of the tumors was also captured in real time. At 24 h after treatment, one mouse in each group was sacrificed and the tumors were harvested, sliced, and stained with H&E and CD31. For long-term observation, the mice body weights and tumor sizes were recorded every two days for a total of 14 days.

### Histological Examinations and Blood Panels

Twenty mice were randomly divided into four groups (n=5). Three groups of mice were intravenously injected with 200 μL NP solution with 3 mg mL^-1^Ti, while the mice in control group were intravenously injected with 200 μL PBS. For the first three groups, the mice were sacrificed 1, 7, and 30 days after injection. Blood was collected in anticoagulant-containing tubes for hematological analysis and the livers, hearts, lungs, spleens, and kidneys were collected for ICP-AES and H&E staining.

## Supplementary Material

Additional characterization and results: TEM images, elemental maps, FTIR spectra, photographs, hydration radius, UV-vis-NIR absorbance spectra, ultraphonic and photoacoustic images and H&E staining; Supplementary Figures S1-S8.

## Figures and Tables

**Table 1:** Parameters and fitted formulas for *in vitro* radiation therapy with and without WTO NPs.

	D_0_	D_q_	SF_2_	SER10	fitted formula
**RT**	4.520	2.483	0.799	-	SF=1-(1-e^-0.4186*D^)^2.828^
**RT + WTO**	2.907	0.808	0.533	1.323	SF=1-(1-e^-0.4427*D^)^1.430^

## Abbreviations

PEG: polyethylene glycol
WTO: W-doped TiO_2_
NPs: nanoparticles
NIR-II: the second near-infrared
RT: Radiotherapy
PTT: photothermal therapy
NIR-I: the first NIR window
MPEs: maximum permissible exposures
PAI: photoacoustic imaging
UV: ultraviolet
CT: computed tomography
XRD: X-ray powder diffraction
TEM: transmission electron microscopy
HRTEM: hgih resolution transmission electron microscopy
XPS: X-ray photoelectron Spectroscopy
UV-vis: ultraviolet-visible
EPR: the electron paramagnetic resonance
DEPE-PEG5000: 1,2-distearoyl-sn-glycero-3-phosphoethanolamine-polyethylene glycol-5000
FTIR: fourier-transform infrared spectroscopy
CCK-8: cell counting kit-8
H&E: hematoxylin and eosin
A/G: albumin/globin ratio
BUN: blood urea nitrogen
RBC: red blood cell
WBC: white blood cell
HCT: hematocrit
HGB: hemoglobin
MCHC: mean corpuscular hemoglobin concentration
MCV: mean corpuscular volume
MCH: mean corpuscular hemoglobin
PLT: platelet count
AST: aspartate aminotransferase
ALP: alkaline phosphatase
ALT: alanine aminotransferase
ICP-MS: inductively coupled plasma-mass spectrometer
PE: plating efficiency
SF: surviving fraction
SER: sensitizer enhancement ratio
PBS: phosphate buffer saline
