# Supplementary material for: W-doped TiO2 nanoparticles with strong absorption in the NIR-II window for photoacoustic/CT dual-modal imaging and synergistic thermoradiotherapy of tumors
Source: Theranostics. 2019 Jul 9;9(18):5214–26. doi: 10.7150/thno.33574 (PMC6691582; doi:10.7150/thno.33574)
Supplement: Supplementary file 1 — Additional characterization and results: TEM images, elemental maps, FTIR spectra, photographs, hydration radius, UV-vis-NIR absorbance spectra, ultraphonic and photoacoustic images and H&E staining; Supplementary Figures S1-S8. [file thnov09p5214s1.pdf]

Supporting Information

W-doped TiO_2 nanoparticles with strong absorption in the NIR-II window for photoacoustic/CT dual-modal imaging and synergistic thermoradiotherapy of tumors

Ke Gao, Wenzhi Tu, Xujiang Yu, Farooq Ahmad, Xiannan Zhang, Weijie Wu, Xiao An, Xiaoyuan Chen and Wanwan Li**

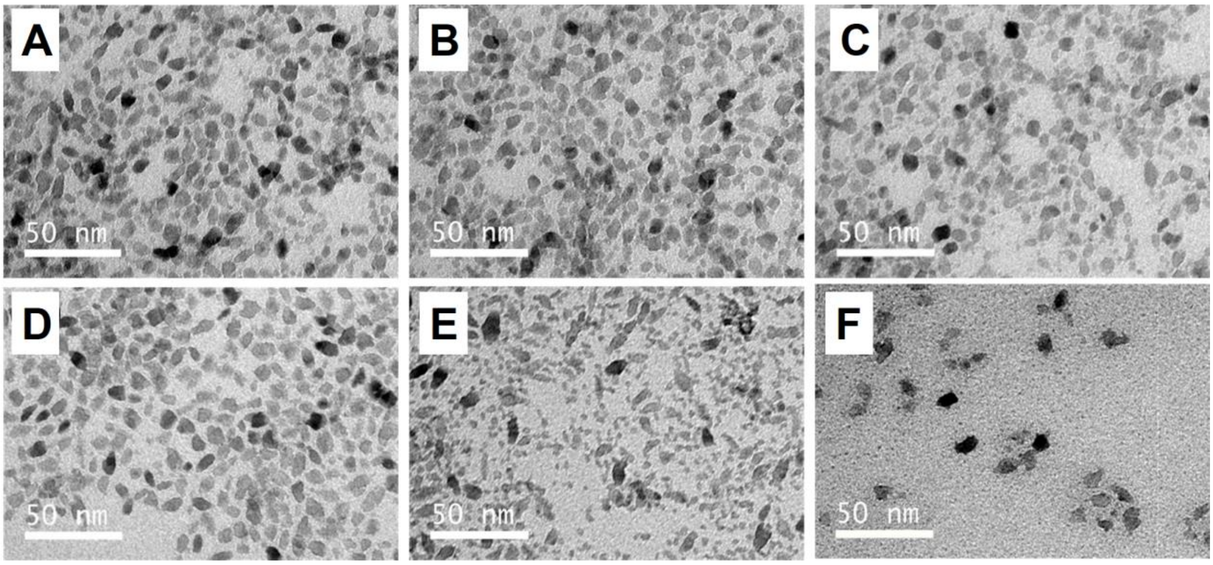

Figure S1. (A-F) TEM images of (A) TiO_2 , (B) TiO_2: 5 at% W, (C)TiO_2: 10 at% W, (D) TiO_2: 15 at% W , (E) TiO_2: 20 at% W NPs and (F) PEGylated TiO_2: 15 at% W NPs.

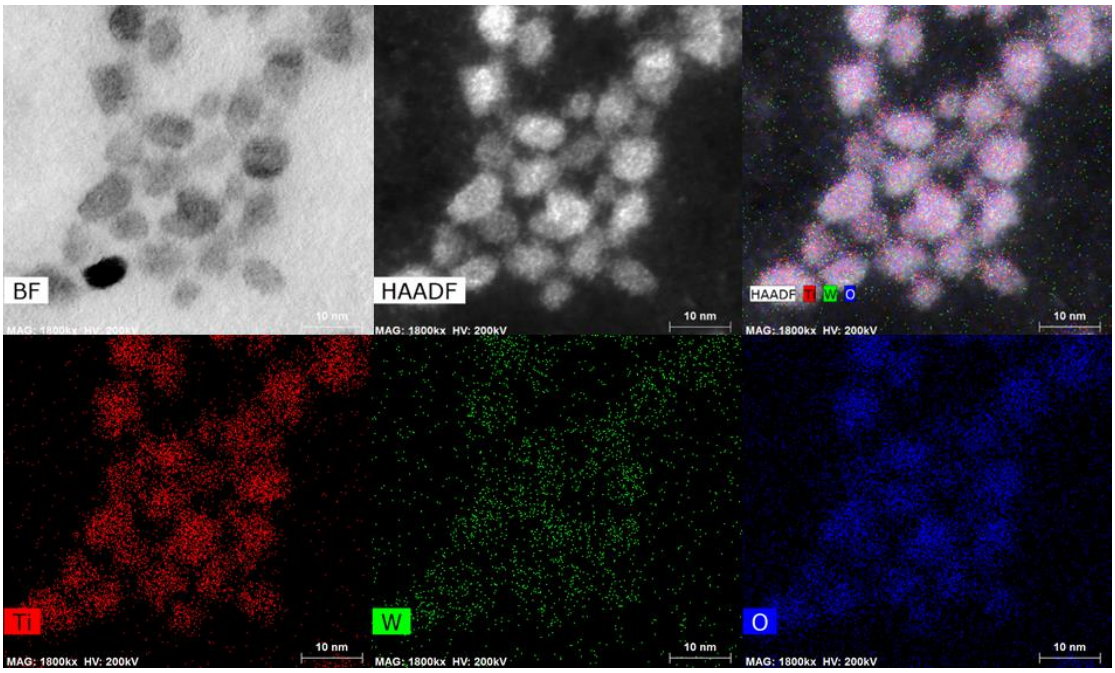

Figure S2. Representative elemental maps of TiO_2: 15 at% W NPs.

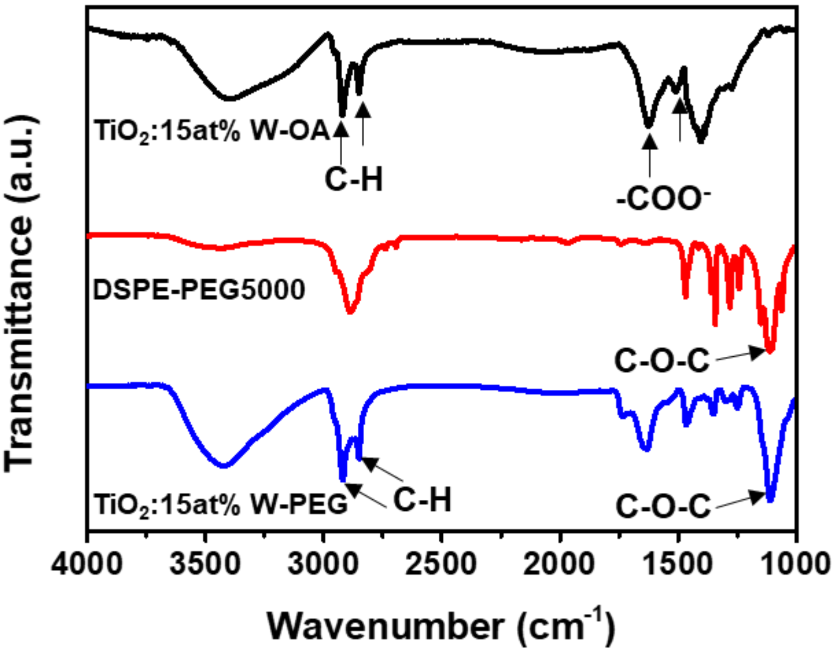

Figure S3. FTIR spectra of TiO_2: 15 at% W-OA, DSPE-PEG_{5000}, and TiO_2: 15 at% W-PEG.

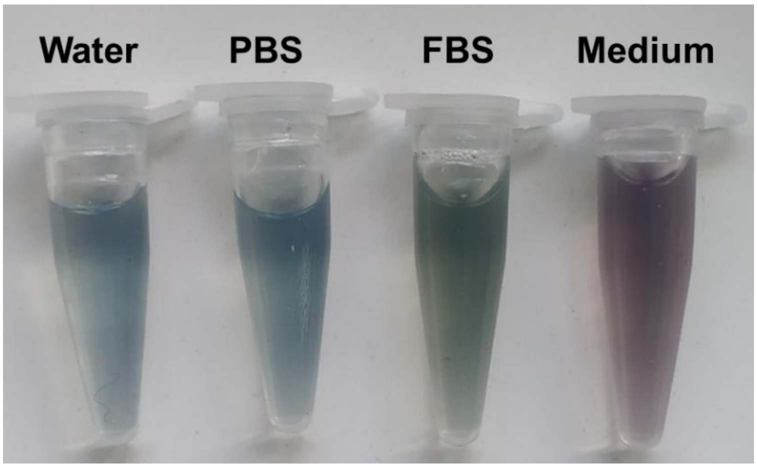

Figure S4. Photograph of PEGylated TiO_2: 15 at% W NPs dispersed in water, PBS, FBS, and cell medium.

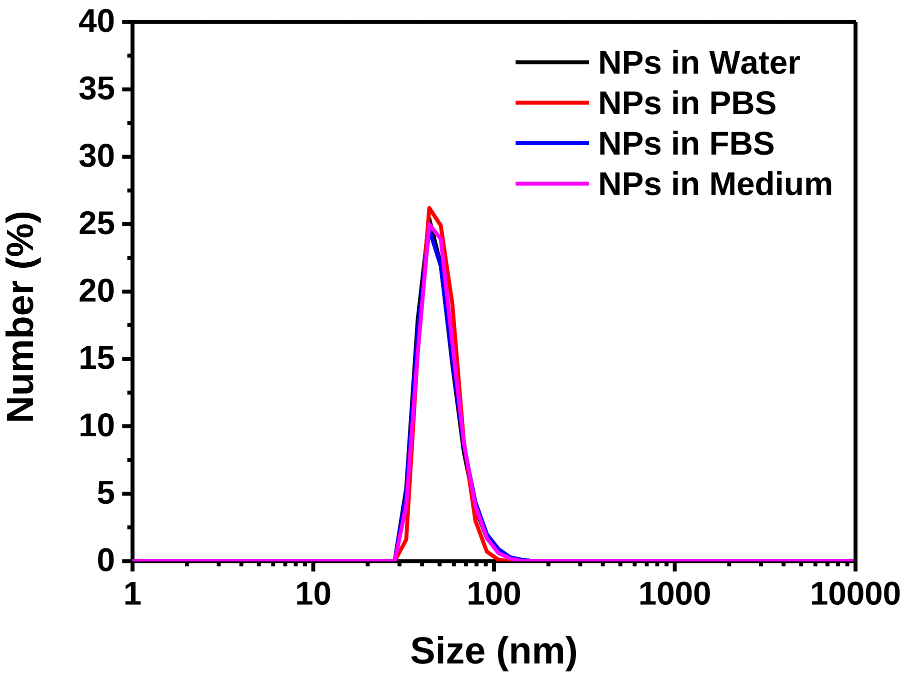

Figure S5. Hydration radius of PEGylated TiO_2: 15 at% W NPs dispersed in water, PBS, FBS and Medium.

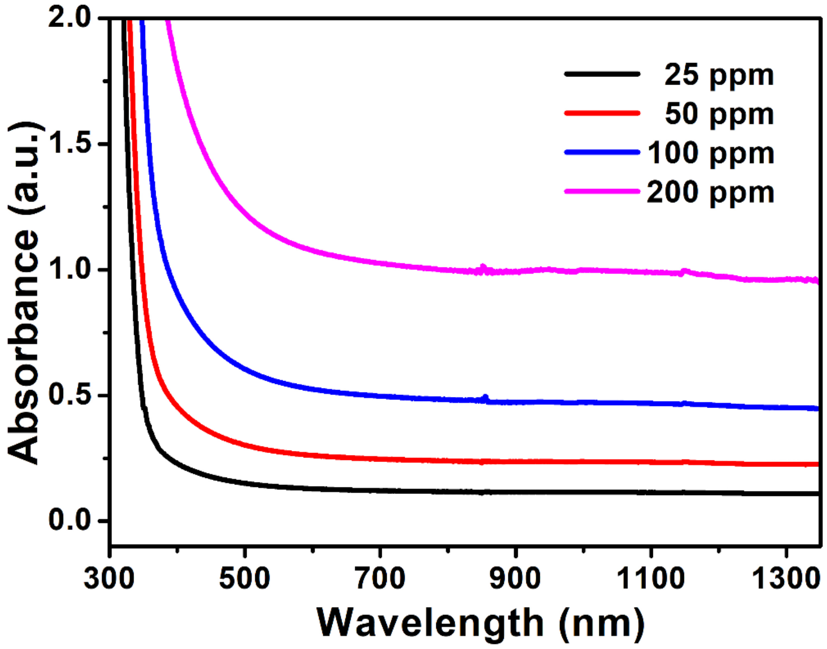

Figure S6. UV-vis-NIR absorbance spectra of PEGylated TiO_2: 15 at% W containing different concentrations of Ti.

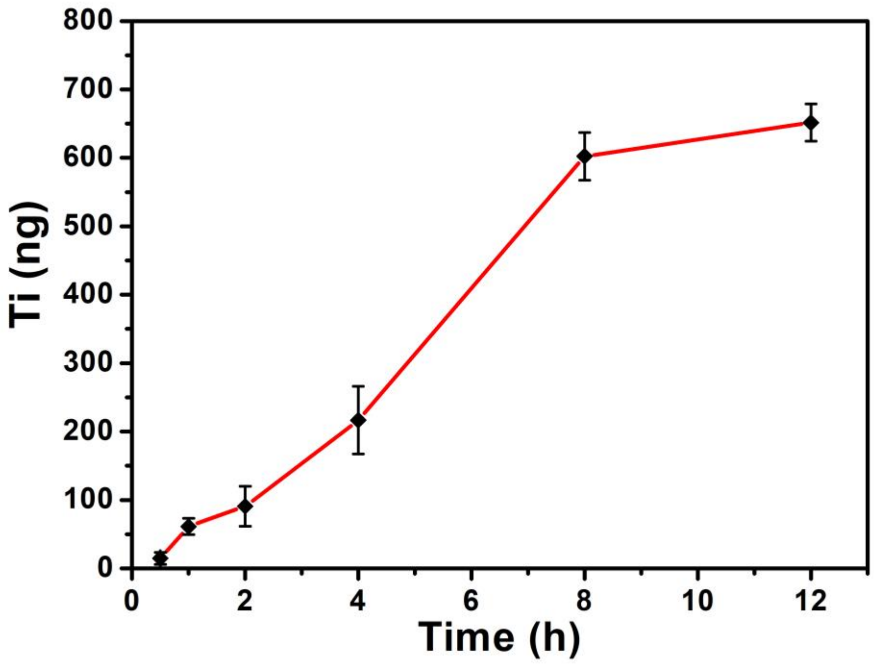

Figure S7. The cellular uptake of PEGylated TiO_2: 15 at% W NPs by 4T1 cells with different internalization time of 0.5, 1, 2, 4, 8 and 12 h.

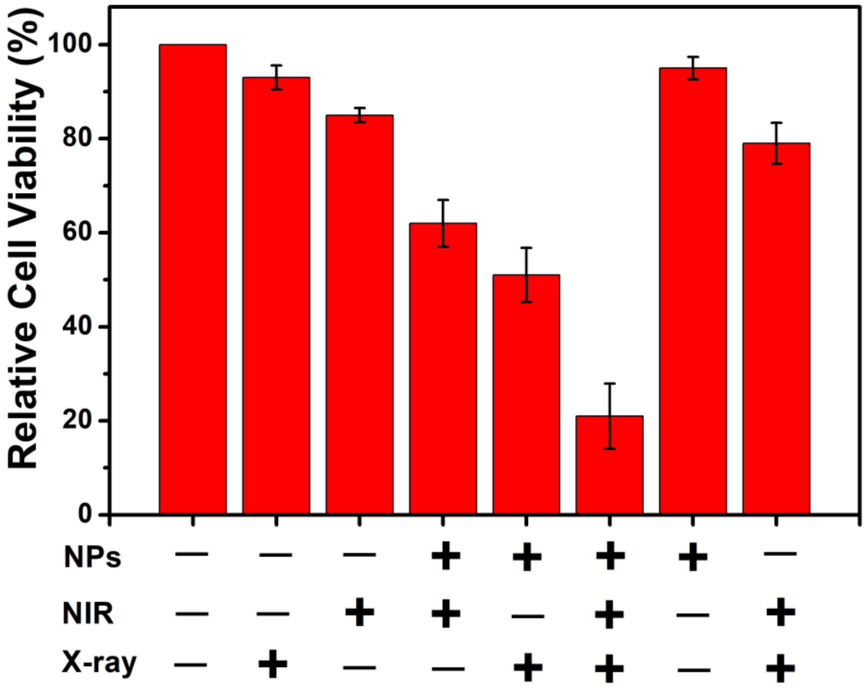

Figure S8. Relative cell viability of each group evaluated using CCK-8 assay.

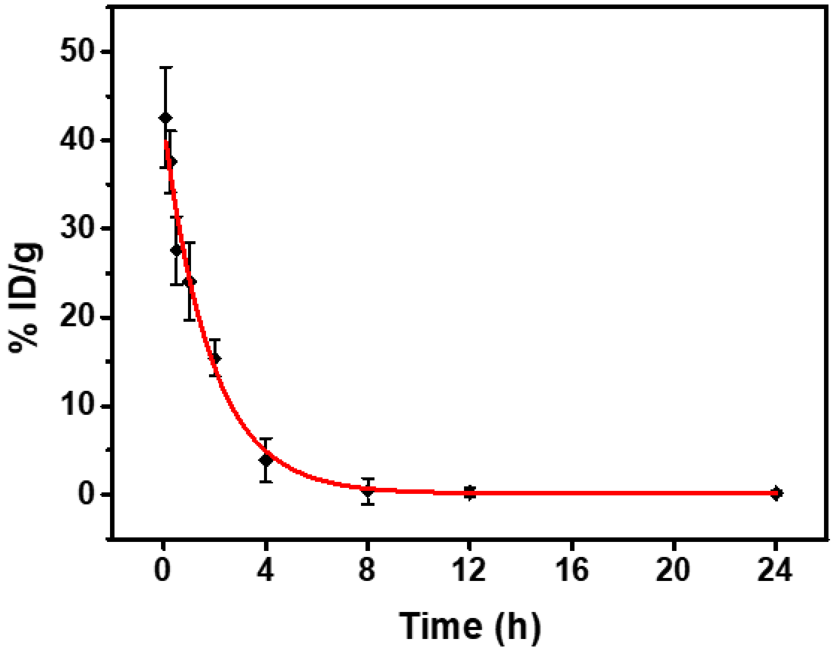

Figure S9. Blood circulation of WTO NPs in mice by detecting the percentage of Ti remaining in the blood among injected dose at different time points.

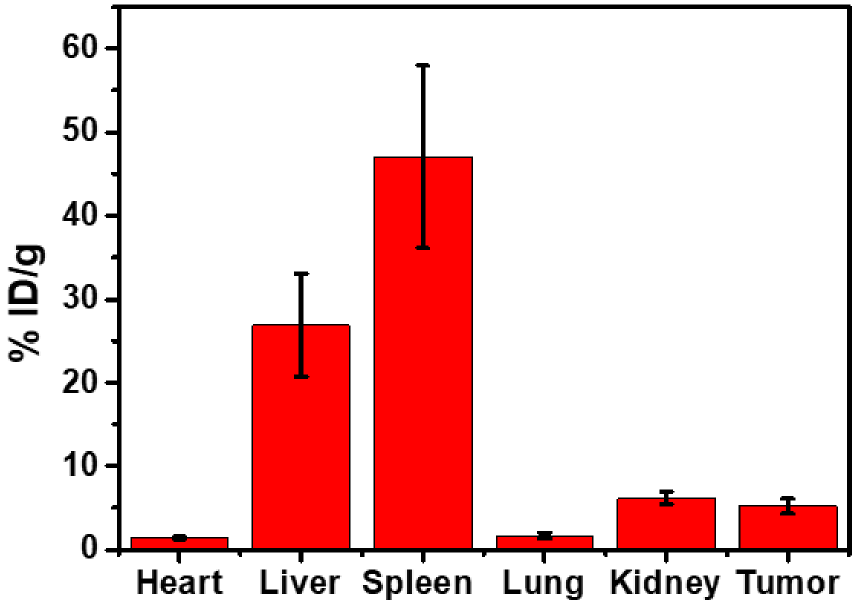

Figure S10. The distribution of WTO NPs in tumor and main organs.

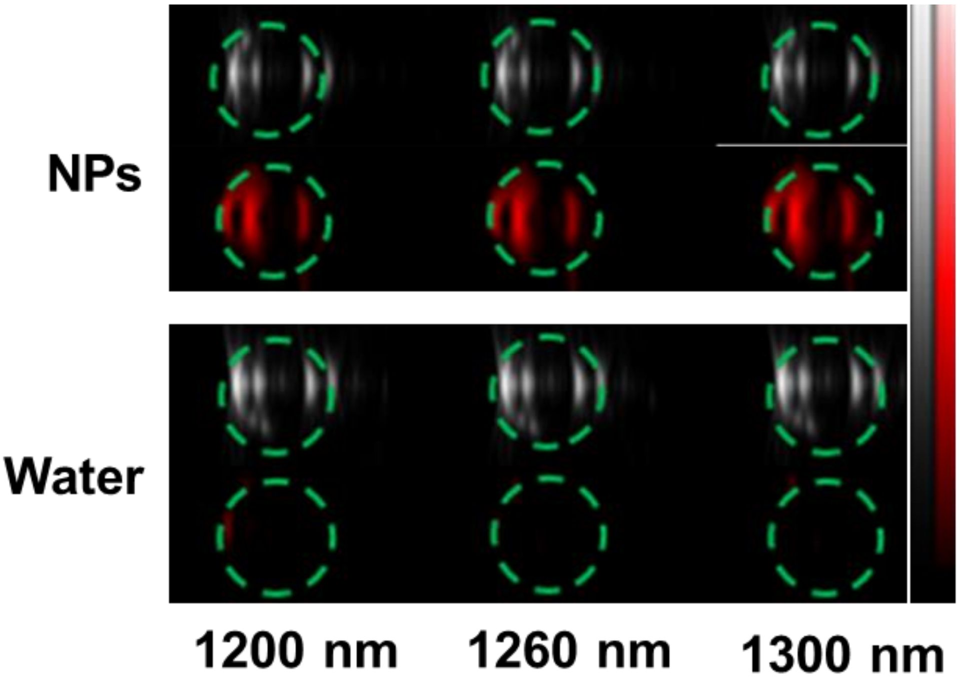

Figure S11. Ultraphonic and photoacoustic images of PEGylated TiO_2: 15 at% W NPs and water in polyurethane microtubes.

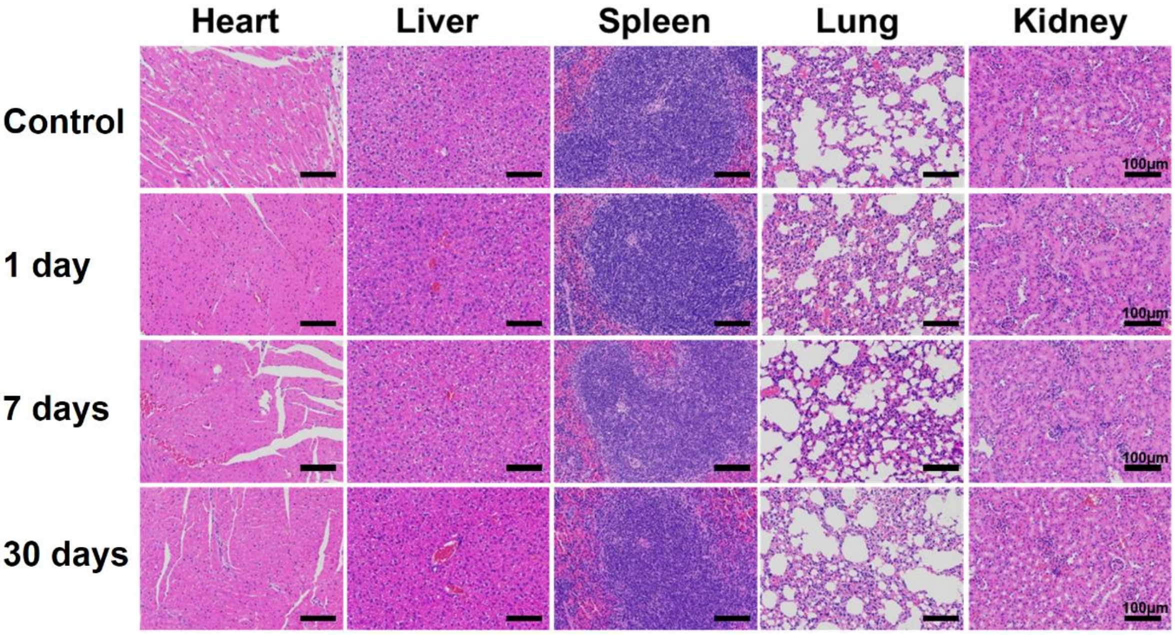

Figure S12. H&E staining of organs from mice sacrificed at different time points.
